# *Alu* and *LINE-1* Hypomethylation Is Associated with HER2 Enriched Subtype of Breast Cancer

**DOI:** 10.1371/journal.pone.0100429

**Published:** 2014-06-27

**Authors:** So Yeon Park, An Na Seo, Hae Yoen Jung, Jae Moon Gwak, Namhee Jung, Nam-Yun Cho, Gyeong Hoon Kang

**Affiliations:** 1 Department of Pathology, Seoul National University College of Medicine, Jongno-gu, Seoul, Korea; 2 Department of Pathology, Seoul National University Bundang Hospital, Bundang-gu, Seongnam, Gyeonggi, Korea; 3 Laboratory of Epigenetics, Cancer Research Institute, Seoul National University, Jongno-gu, Seoul, Korea; University of Bonn, Institut of experimental hematology and transfusion medicine, Germany

## Abstract

The changes in DNA methylation status in cancer cells are characterized by hypermethylation of promoter CpG islands and diffuse genomic hypomethylation. *Alu* and long interspersed nucleotide element-1 (*LINE-1*) are non-coding genomic repetitive sequences and methylation of these elements can be used as a surrogate marker for genome-wide methylation status. This study was designed to evaluate the changes of *Alu* and *LINE-1* hypomethylation during breast cancer progression from normal to pre-invasive lesions and invasive breast cancer (IBC), and their relationship with characteristics of IBC. We analyzed the methylation status of *Alu* and *LINE-1* in 145 cases of breast samples including normal breast tissue, atypical ductal hyperplasia/flat epithelial atypia (ADH/FEA), ductal carcinoma in situ (DCIS) and IBC, and another set of 129 cases of IBC by pyrosequencing. *Alu* methylation showed no significant changes during multistep progression of breast cancer, although it tended to decrease during the transition from DCIS to IBC. In contrast, *LINE-1* methylation significantly decreased from normal to ADH/FEA, while it was similar in ADH/FEA, DCIS and IBC. In IBC, *Alu* hypomethylation correlated with negative estrogen receptor (ER) status, and *LINE-1* hypomethylation was associated with negative ER status, *ERBB2 (HER2)* amplification and p53 overexpression. *Alu* and *LINE-1* methylation status was significantly different between breast cancer subtypes, and the HER2 enriched subtype had lowest methylation levels. In survival analyses, low *Alu* methylation status tended to be associated with poor disease-free survival of the patients. Our findings suggest that *LINE-1* hypomethylation is an early event and *Alu* hypomethylation is probably a late event during breast cancer progression, and prominent hypomethylation of *Alu* and *LINE-1* in HER2 enriched subtype may be related to chromosomal instability of this specific subtype.

## Introduction

Common epigenetic changes in cancer include CpG island hypermethylation of gene promoters and genome-wide hypomethylation of non-coding genomic regions. While promoter CpG island hypermethylation is an alternative mechanism for inactivating tumor suppressor genes, resulting in their transcriptional silencing [1], [2], genome-wide hypomethylation is associated with genomic instability and hence facilitates tumor progression [3], [4]. In breast cancer, promoter CpG island hypermethylation has been described for genes involved in all aspects of cellular function and was found to be associated with various histopathologic characteristics, including tumor grade [5], [6], hormone receptor [7], [8], HER2/neu status [9] and molecular subtype [10]–[13]. However, few studies have focused on genome-wide hypomethylation in breast cancer and its association with the clinicopathologic characteristics of breast cancer.

Genome-wide global hypomethylation affects repetitive transposable DNA elements and they reside mainly in the intergenic and intronic regions of the genome [14], [15]. *Alu* and long interspersed nucleotide element-1 (*LINE-1*) are major components of repetitive transposable DNA elements, constituting approximately 17% and 11% of the human genome [14]. Because of their high frequencies in the genome, *Alu* and *LINE-1* methylation status serve as a useful surrogate marker for genome-wide methylation status. In normal cells, CpG sites within *Alu* and *LINE-1* are usually methylated, thus maintaining transcriptional inactivation and inhibiting retrotransposition [16]. However, hypomethylation of *Alu* and *LINE-1* is consistently found in many types of human cancers [17]–[23]. Hypomethylation of transposable elements such as *Alu* and *LINE-1* causes transcriptional activation, resulting in retrotransposition of the transposable element, chromosome alteration and thus genomic instability [18], [24]. *Alu* and *LINE-1* hypomethylation have been reported as early events in the multistep carcinogenesis of colorectal cancer [17], [20], [22]. However, some controversies exist in other types of cancer [18], [19], [21]. Moreover, in breast cancer, studies on changes of *Alu* and *LINE-1* methylation status during the multistep progression of breast cancer have been rare.

Comprehensive gene expression profiling has identified five major molecular subtypes in breast cancer including luminal A, luminal B, HER2+, basal-like, and normal breast-like subtype [25]–[27]. Array-based comprehensive DNA methylation profiling has shown that breast cancer molecular subtypes have their own methylation profiles [10]–[13]. Interestingly, these different methylation profiles were found throughout the CpG islands of the genome, not limited to functional genes [12]. In a previous study, we reported that promoter CpG island methylation was significantly lower in the basal-like subtype of breast cancer than in the other subtypes, and the methylation of promoter CpG islands was inversely related to stem cell phenotypes as revealed by CD44+/CD24− and ALDH1 expression [28]. Thus, there are possibilities that *Alu* and *LINE-1* hypomethylation may be different according to breast cancer subtype or CD44+/CD24− and ALDH1+ breast cancer stem cell (BCSC) phenotypes.

In this study, we evaluated *Alu* and *LINE-1* hypomethylation during breast cancer progression from normal epithelium to pre-invasive lesions [atypical ductal hyperplasia (ADH), flat epithelial atypia (FEA) and ductal carcinoma in situ (DCIS)] and invasive breast cancer (IBC) for the first step, and then assessed *Alu* and *LINE-1* hypomethylation in another set of IBC and correlated them with characteristics of IBC. We investigated in particular whether *Alu* and *LINE-1* hypomethylation is distinct in relation to breast cancer subtype and breast cancer stem cell phenotypes as represented by CD44+/CD24− and ALDH1 expression.

## Materials and Methods

### Ethics statement

This study was approved by the institutional review board of Seoul National University Bundang Hospital (protocol # B-1005-100-302). The requirements for informed consent from participants were waived by the institutional review board as all the specimen were previously collected for pathologic examination after surgery and all the data were analyzed anonymously.

### Patients and tissue specimens

We used 2 different sets of breast tissue samples in this study. The first set was used to assess the changes of *Alu* and *LINE-1* hypomethylation during multistep breast cancer progression and included 145 breast samples from one of our previous studies [6], including 30 of normal breast tissue, 30 ADH/FEA (20 ADH and 10 FEA), 35 pure DCIS, and 50 IBCs. The second set of 179 cases was composed of 129 cases of IBC in another previous study [28] and 50 cases of IBC from the first set, was used to evaluate the characteristics of IBC associated with *Alu* and *LINE-1* hypomethylation. Clinicopathologic characteristics of 179 patients with IBC are summarized in Table 1.

**Table 1:** Clinicopathologic features of 179 invasive breast cancers.

Characteristics	No. (%)
Age, yrs.	
Mean	50.8
Range	20 to 85
T stage	
T1	76 (42.5)
T2	99 (55.3)
T3	4 (2.2)
N stage	
N0	105 (58.7)
N1	48 (26.8)
N2	12 (6.7)
N3	14 (7.8)
Histologic grade	
I	16 (8.9)
II	49 (27.4)
III	114 (63.7)
Lymphatic invasion	
Absent	99 (55.3)
Present	80 (44.7)
Venous invasion	
Absent	172 (96.1)
Present	7 (3.9)
P53 overexpression	
Absent	117 (65.4)
Present	62 (34.6)
Ki-67	
<20%	68 (38.0)
≥20%	111 (62.0)
ER	
Negative	82 (45.8)
Positive	97 (54.2)
PR	
Negative	97 (54.2)
Positive	82 (45.8)
HER2	
Negative	109 (60.9)
Positive	70 (39.1)
Subtype	
Luminal A	36 (20.1)
Luminal B	33 (18.4)
Luminal-HER2	30 (16.8)
HER2 enriched	40 (22.3)
Basal-like	40 (22.3)

### Immunohistochemistry and definition of breast tumor subtypes

Expression of standard biomarkers including estrogen receptor (ER), progesterone receptor (PR), HER2, Ki-67, cytokeratin5/6, and EGFR and BCSC markers (ALDH1, CD44 and CD24) had been assessed previously [6], [28], and therefore were used in this study with the same cutoff values. In IBC, breast cancer subtypes were classified into 5 groups according to the definition by Voduc et al. [29] as follows: luminal A (ER+ or PR+, HER2−, Ki-67 <14%), luminal B (ER+ or PR+, HER2−, Ki-67 ≥14%), luminal-HER2 (ER+ or PR+, HER2+), HER2 enriched (ER−, PR−, HER2+), basal-like (ER−, PR−, HER2−, cytokeratin 5/6+ or EGFR+). The 179 cases consisted of 36 luminal A, 33 luminal B, 30 luminal-HER2, 40 HER2 enriched, and 40 basal-like subtypes.

### *Alu* and *LINE-1* methylation

Remnant DNA samples from previous studies [6], [28] were used for the analysis of *Alu* and *LINE-1* methylation. Otherwise, slides were reviewed and representative area was manually dissected under microscope using three to five serial sections (4 µm thick) stained with hematoxylin and eosin. Dissected tissues were subjected to tissue lysis using proteinase K lysis buffer containing 0.5% Tween 20 (Sigma, St. Louis, MO, USA), 100 mM of Tris HCl buffer (pH 7.6), 1 mM of EDTA, and 1 mg/ml of proteinase K (Sigma) at 55°C for 24 h to 48 h. Bisulfite modification of the digested samples was performed using an EZ DNA methylation kit (Zymo Research, Orange, CA, USA) according to manufacturer's protocols. Methylation levels of *Alu* and *LINE-1* were measured using a pyrosequencing methylation assay. PCR and subsequent pyrosequencing for each gene were carried out using the PyroMark kit (Qiagen, Valencia, CA, USA). In order to monitor plate-to-plate variability, we included highly methylated DNA (in vitro methylated DNA) and unmethylated DNA (whole genome-amplified DNA) controls in each plate and measured *Alu* or *LINE-1* methylation levels of these two controls. We confirmed the *Alu* or *LINE-1* methylation levels of these control DNAs were within 2 S. D. ranges from the corresponding mean value which were determined after measurement of *Alu* or *LINE-1* methylation levels sixty times repeat. To minimize plate-to-plate variability, we also conducted randomization of the order in which the study samples were subjected to pyrosequencing methylation analysis.

The oligonucleotide primers and PCR conditions are described in Table 2. PCR primers, designed for a consensus sequence for *Alu* or *LINE-1*, amplified a global pool of *Alu* or *LINE-1* rather than a single element or genomic locus. The biotinylated PCR products were purified and quantified in the PyroMark Q24 System (Biotage AB, Uppsala, Sweden) according to the procedure described previously. Briefly, PCR was carried out in a 25-µL PCR reaction containing 2-µL bisulfite-treated genomic DNA, 60 mM Tris-HCI (pH 8.8), 15 mM ammonium sulfate, 0.5 mM MgCl_2_, 1 mM dNTP mix, and 1 U of Taq polymerase. The PCR cycling condition was as follows: 45 cycles of 95°C for 20 seconds, 50°C for 20 seconds, 72°C for 20 seconds, and 72°C for 5 minutes. The amounts of G relative to the sum of G and A at each CpG site were analyzed for *Alu* methylation. The *Alu* methylation level measured the percentages of methylcytosine at 4 CpG sites. The amount of C relative to the sum of C and T at each CpG site was calculated as a percentage. The average of the relative amounts of C in the 4 CpG sites was taken as the overall *LINE-1* methylation level in a given tumor [18].

**Table 2:** Sequences of *Alu* and *LINE-1*.

Gene	Primer		Temperature (°C)
*Alu*	Forward	5′-biotin-TTAAAAATATAAAAATTAGT-3′	54, 52, 50, 48
	Reverse	5′-CCAAACTAAAATACAATAA-3′	(step down)
	Sequencing	5′-AATAACTAAAATTACAAAC-3′	
*LINE-1*	Forward	5′-TTTTGAGTTAGGTGTGGGATATA-3′	52
	Reverse	5′-biotin-AAAATCAAAAAATTCCCTTTC-3′	
	Sequencing	5′-AGTTAGGTGTGGGATATAGT-3′	

Although PCR-based assay is known to be highly sensitive even in poor-quality clinical DNA samples such as paraffin-embedded section [30], we determined the adequacy of formalin-fixed, paraffin-embedded section for the pyrosequencing methylation assay. *LINE-1* methylation levels were assessed for 20 paired snap-frozen and formalin-fixed paraffin-embedded tissue samples, including 10 normal lymph nodes, 5 colon cancers, and 5 breast cancers. Correlation analysis revealed a strong, positive linear correlation between two measures (Pearson correlation coefficient, 0.928; *P*<0.001) (Figure S1), indicating the suitability of formalin-fixed, paraffin-embedded section for the assay.

### Statistical analysis

All statistical analyses were performed using the software package Statistical Package for Social Sciences Version 18.0 (SPSS Inc., Chicago, IL, USA). When comparing *Alu* and *LINE-1* methylation levels among three or more groups, the non-parametric Kruskal-Wallis test was performed. The differences in *Alu* and *LINE-1* methylation levels between two groups were analyzed using the Mann-Whitney U test, and the corrections for multiple testing were performed with Bonferroni method, if indicated. The comparison of decrease in *Alu* and *LINE-1* methylation levels during multistep breast cancer progression was evaluated using a linear trend test (*P*_trend_). The correlation between *Alu* and *LINE-1* methylation levels was evaluated using the Pearson correlation analysis. In IBC, median values for *Alu* and *LINE-1* methylation levels were used as cutoff values, and IBCs were divide into methylation and hypomethylation groups, and the chi-squared test or Fisher's exact test was used to determinate the association between methylation levels of *Alu* and *LINE-1* and clinicopathologic features of IBCs. Kaplan-Meier survival curves for disease-free survival were plotted for *Alu* and *LINE-1* (methylation vs. hypomethylation using the median as the cutoff value) and the significance of differences between the two groups was determined using the log-rank test. All tests were two-tailed and statistical significance was set as *P* values <0.05.

## Results

### *Alu* and *LINE-1* methylation levels during breast cancer progression

**Figure 1:**
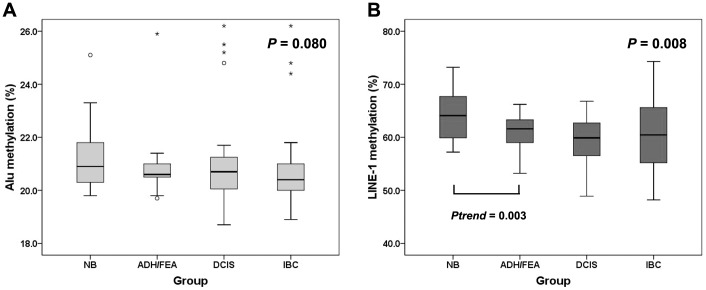
Box plot illustrating the methylation levels of *Alu* and *LINE-1* in normal breast (NB), atypical ductal hyperplasia (ADH)/flat epithelial atypia (FEA), ductal carcinoma in situ (DCIS), and invasive breast cancer (IBC). (A) *Alu* methylation levels are not different among the four groups of breast lesions. (B) *LINE-1* methylation levels are significantly different between four groups of breast lesions, and significantly decrease during transition from normal breast to ADH/FEA. The box shows the first to third quartiles, the horizontal line inside the box represents the median, the whiskers extend to minimum and maximum values within 1.5 times the interquartile range (IQR) from the first and third quartiles. Outliers are represented by small circles and, extreme values (more than 3 times IQR), by asterisks.

To examine the changes of *Alu* and *LINE-1* methylation levels during multistep progression of breast cancer, we compared their methylation levels in normal breast, ADH/FEA, DCIS, and IBC. The median of *Alu* methylation levels in normal breast, ADH/FEA, DCIS, and IBC were 20.9%, 20.6%, 20.7%, and 20.4%, respectively. *Alu* methylation levels were not different among the 4 groups of breast lesions (Kruskal-Wallis test; *P* = 0.080). In addition, there were no significant changes during the stepwise progression of breast cancer (from normal breast to ADH/FEA, *P*_trend_ = 0.284; from ADH/FEA to DCIS, *P*_trend_ = 0.929; from DCIS to IBC, *P*_trend_ = 0.096), although *Alu* methylation tended to decrease during the transition from DCIS to IBC (Figure 1A). The median *LINE-1* methylation levels in normal breast, ADH/FEA, DCIS, and IBC were 64.1%, 61.6%, 59.9%, and 61.6%, respectively, being significantly different among the 4 groups (Kruskal-Wallis test; *P* = 0.008). As shown in Figure 1B, *LINE-1* methylation levels were significantly decreased during progression from normal to ADH/FEA (*P*_trend_ = 0.003). However, there were no further decreases from ADH/FEA to DCIS (*P*_trend_ = 0.151) and from DCIS to IBC (*P*_trend_ = 0.386).

### Associations of *Alu* and *LINE-1* methylation levels with clinicopathologic features of IBC

To evaluate the characteristic of IBC associated with *Alu* and *LINE-1* methylation status, we analyzed *Alu* and *LINE-1* methylation levels in the 179 IBC cases. The median *Alu* and *LINE-1* methylation levels were 20.2% (interquartile range: 19.8 to 20.8%) and 59.4% (interquartile range: 54.9 to 64.6%), respectively. *Alu* methylation levels positively correlated with *LINE-1* methylation levels (Pearson correlation coefficient, *r* = 0.288, *P*<0.001). The associations between *Alu* and *LINE-1* methylation levels and clinicopathologic features of tumor are summarized in Table 3. *Alu* hypomethylation was associated with ER negativity (*P* = 0.006). *LINE-1* hypomethylation was significantly associated with ER negativity (*P*<0.001), PR negativity (*P* = 0.011), *ERBB2 (HER2)* amplification (*P* = 0.006) and p53 overexpression (*P* = 0.028). However, there were no significant differences in *Alu* and *LINE-1* methylation levels in relation to patients' age, T stage, lymph node metastasis, histologic grade, lymphovascular invasion and Ki-67 proliferation index.

**Table 3:** Associations of *Alu* and *LINE-1 methylation status with* clinicopathologic features of invasive breast cancer.

Characteristics	*Alu*		*P value*	*LINE-1*		*P value*
	Hypomethylated (<20.2%)	Methylated (≥20.2%)		Hypomethylated (<59.4%)	Methylated (≥59.4%)	
Age			0.454			0.136
<50 years	42 (55.3)	51 (49.5)		41 (46.1)	52 (57.8)	
≥50 years	34 (44.7)	52 (50.5)		48 (53.9)	38 (42.2)	
T stage			0.446			0.547
T1	35 (46.1)	41 (39.8)		40 (44.9)	36 (40.0)	
T2 & T3	41 (53.9)	62 (60.2)		49 (55.1)	54 (60.0)	
N stage			0.446			0.173
N0	42 (55.3)	63 (61.2)		57 (64.0)	49 (53.3)	
N1–N3	34 (44.7)	40 (38.8)		32 (36.0)	42 (46.7)	
Histologic grade			0.640			0.214
Low (I & II)	26 (34.2)	39 (37.9)		28 (31.5)	37 (41.1)	
High (III)	50 (65.8)	64 (62.1)		61 (68.5)	53 (58.9)	
Lymphatic invasion			0.366			0.453
Absent	39 (51.3)	60 (58.3)		52 (58.4)	47 (52.2)	
Present	37 (48.7)	43 (41.7)		37 (41.6)	43 (47.8)	
Venous invasion			0.460			0.444
Absent	72 (94.7)	100 (97.1)		87 (97.8)	85 (94.4)	
Present	4 (5.3)	3 (2.9)		2 (2.2)	5 (5.6)	
P53 overexpression			0.429			0.028
Negative	47 (61.8)	70 (68.0)		51 (57.3)	66 (73.3)	
Positive	29 (38.2)	33 (32.0)		38 (42.7)	24 (26.7)	
Ki-67			0.437			0.442
<20%	26 (34.2)	42 (40.8)		31 (34.8)	37 (41.1)	
≥20%	50 (65.8)	61 (59.2)		58 (65.2)	53 (58.9)	
ER			0.006			<0.001
Negative	44 (57.9)	38 (36.9)		54 (60.7)	28 (31.1)	
Positive	32 (42.1)	65 (63.1)		35 (39.3)	62 (68.9)	
PR			0.095			0.011
Negative	47 (61.8)	50 (48.5)		57 (64.0)	40 (44.4)	
Positive	29 (38.2)	53 (51.5)		32 (36.0)	50 (55.6)	
*ERBB2* amplification			0.354			0.006
Negative	43 (56.6)	66 (64.1)		45 (50.6)	64 (71.1)	
Positive	33 (43.4)	37 (35.9)		44 (49.4)	26 (28.9)	

### Differences in *Alu* and *LINE-1* methylation levels according to breast cancer subtypes

**Figure 2:**
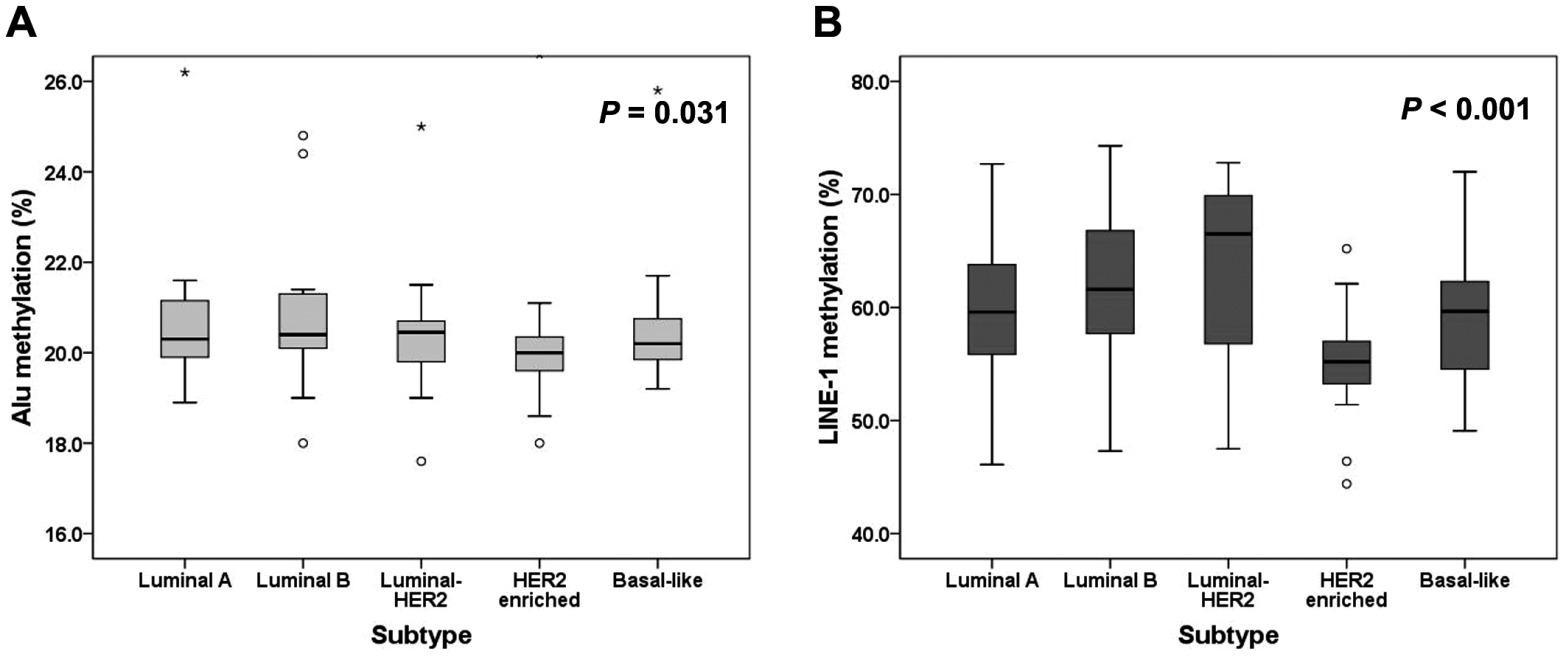
Box plot depicting the methylation levels of *Alu* and *LINE-1* in the subtypes of breast cancer. The methylation levels of *Alu* (A) and *LINE-1*(B) are lowest in the HER2 enriched subtype. The box shows the first to third quartiles, the horizontal line inside the box represents the median, the whiskers extend to minimum and maximum values within 1.5 times the interquartile range (IQR) from the first and third quartiles. Outliers are represented by small circles and, extreme values (more than 3 times IQR), by asterisks.

We also compared the methylation levels of *Alu* and *LINE-1* according to breast cancer subtype in the 179 IBCs. The *Alu* and *LINE-1* methylation levels were significantly different among the subtypes (Kruskal-Wallis test; *P* = 0.031, *P*<0.001, respectively, Figure 2). *Alu* methylation levels were lowest in the HER2 enriched subtype, showing significant difference with luminal B subtype (Mann-Whitney U test; *P* = 0.003). Similarly, *LINE-1* methylation levels were lowest in the HER2 enriched subtype, being significantly lower than those in luminal A, luminal B, luminal-HER2, and basal-like subtypes (HER2 enriched vs. luminal A, HER2 enriched vs. luminal B, HER2 enriched vs. luminal-HER2, *P*<0.001; HER2 enriched vs. basal-like, *P* = 0.001).

### Association of *Alu* and *LINE-1* methylation levels with BCSC phenotypes

**Figure 3:**
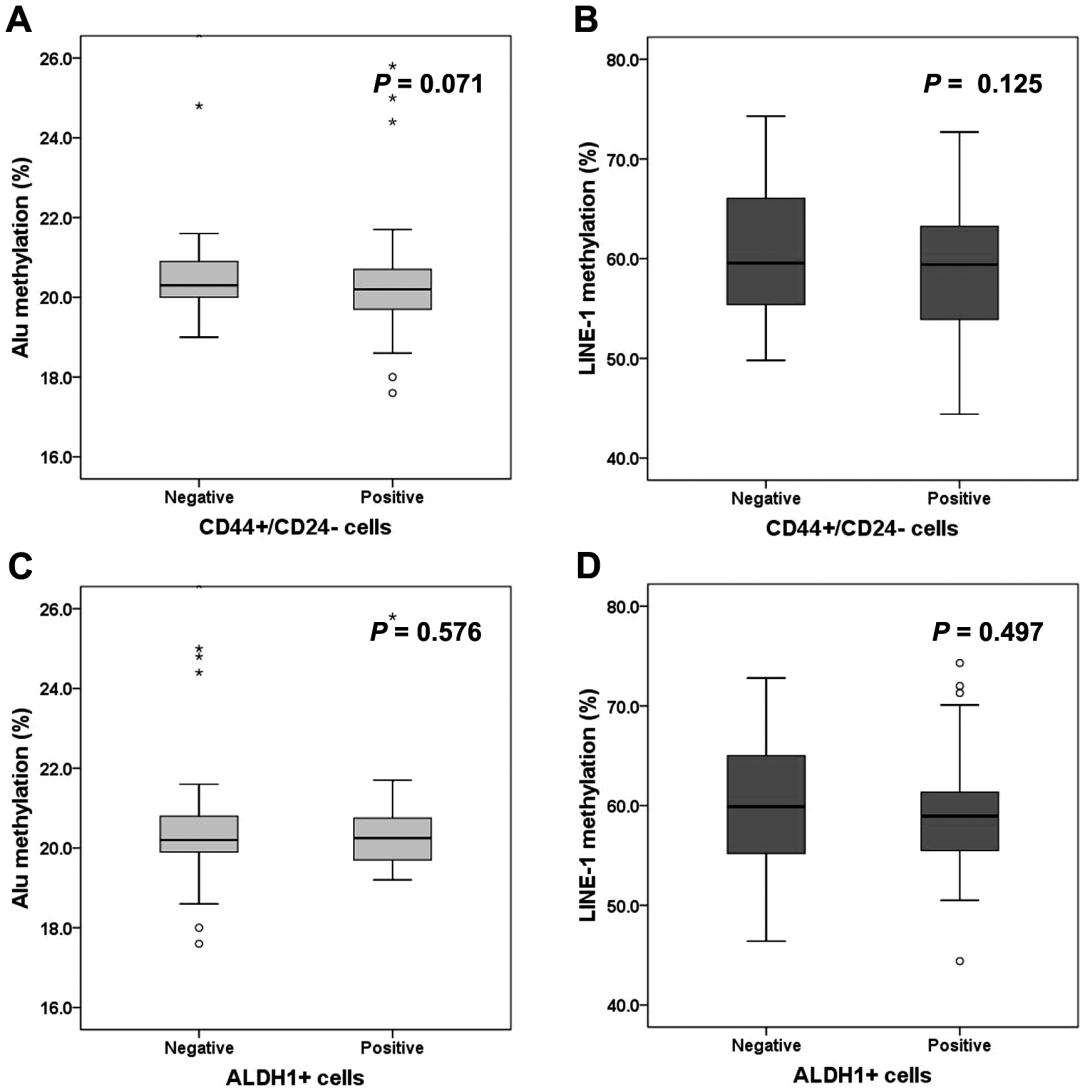
Comparison of the methylation levels of *Alu* and LINE-1 in relation to CD44+/CD24− and ALDH1 expression. CD44+/CD24− phenotype tends to be associated with lower *Alu* (A) and *LINE-1* (B) methylation levels. ALHD1 expression is not related to *Alu* (C) or *LINE-1* (D) methylation levels. The box shows the first to third quartiles, the horizontal line inside the box represents the median, the whiskers extend to minimum and maximum values within 1.5 times the interquartile range (IQR) from the first and third quartiles. Outliers are represented by small circles and, extreme values (more than 3 times IQR), by asterisks.

To evaluate the relevance of *Alu* or *LINE-1* hypomethylation to BCSC phenotype in IBC, we analyzed methylation levels of *Alu* and *LINE-1* according to BCSC phenotypes in 179 cases of IBC. IBCs with CD44+/CD24− phenotype showed a tendency to have lower *Alu* and *LINE-1* methylation levels than IBCs without CD44+/CD24− phenotype (Mann-Whitney U test; *Alu*, *P* = 0.071; *LINE-1*, *P* = 0.125; Figure 3A and 3B). However, there were no significant differences in *Alu* and *LINE-1* methylation levels in relation to ALDH1 expression (*Alu*, *P* = 0.576; *LINE-1*, *P* = 0.497; Figure 3C and 3D).

### *Alu* and *LINE-1* methylation levels and patient outcomes in IBC

**Figure 4:**
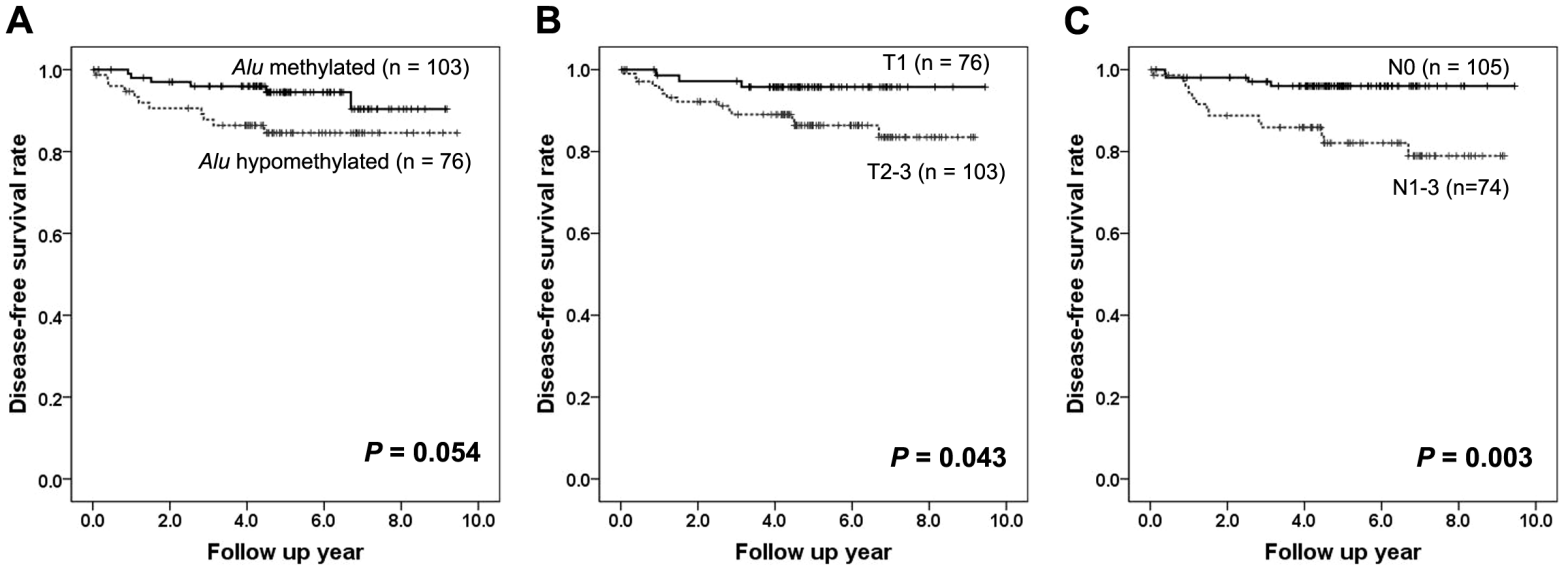
Survival curves according to the methylation levels of *Alu*, pT stage and pN stage in 179 invasive breast cancers. (A) *Alu* hypomethylation tends to be associated with poor disease-free survival. High T stage (B) and nodal metastasis (C) are associated with poor disease-free survival of the patients.

We also investigated the prognostic significance of the *Alu* and *LINE-1* hypomethylation in IBCs. At the time of analysis, the median follow-up after surgery was 4.9 years (range: 0.1 to 9.5 years). There were 5 (3%) loco-regional recurrences, 11 (6%) distant metastases and 1 cancer-related death (0.6%) as first events. In Kaplan-Meier survival analyses, *Alu* hypomethylation (<20.2%) tended to be associated with shorter disease-free survival time (*P* = 0.054; Figure 4A). On the other hand, there were no survival differences with regard to *LINE-1* methylation status (*P* = 0.258). Only T stage (T1 vs. T2–3; *P* = 0.043; Figure 4B) and nodal metastasis (N0 vs. N1–3; *P* = 0.003; Figure 4C) were associated with poor disease-free survival of the patients in this study population.

## Discussion

Genome-wide global hypomethylation is a common epigenetic change in cancer. Moreover, this process is directly correlated with cancer progression [30]. However, whether it progressively evolves or abruptly changes during multistep carcinogenesis has not yet been determined. Furthermore, the pattern of changes in global hypomethylation during multistep carcinogenesis seems to depend on tissue type. Studies have shown that methylation levels in repetitive DNA elements including *Alu* and *LINE-1* decrease gradually during multistep carcinogenesis in hepatocellular carcinoma [21], and extrahepatic cholangiocarcinoma [19]. However, in colorectal cancers, methylation levels of *Alu* or *LINE-1* have been reported to be significantly decreased during transition from normal tissue to adenoma, but not in the progression from adenoma to carcinoma, suggesting *Alu* and *LINE-1* hypomethylation is an early event in colorectal carcinogenesis [17], [20], [22]. In gastric cancer, *LINE-1* methylation decreased during the progression from intestinal metaplasia to gastric adenoma without further decrease in the progression to carcinoma, whereas *Alu* methylation decreased during transition from chronic gastritis to intestinal metaplasia and from gastric adenoma to carcinoma [18]. In the present study about multistep progression of breast cancer, *LINE-1* methylation levels decreased significantly from normal breast to ADH/FEA with no further decreases from ADH/FEA to DCIS or from DCIS to IDC. In the light of this finding, our study suggests that *LINE-1* hypomethylation is an early event during breast cancer progression, in agreement with that of gastric [18] and colorectal cancer [17], [20], [22]. However, *Alu* methylation levels showed no significant difference during multistep progression of breast cancer, although they tended to decrease during the transition from DCIS to IBC.

Recently, van Hoesel et al. [23] studied *LINE-1* methylation index during breast cancer progression from normal breast to ductal hyperplasia, ADH, DCIS and stage I IBC using absolute quantitative assessment of methylated alleles PCR assay and showed the most profound and statistically significant decrease of *LINE-1* methylation index during transition from ADH to DCIS. In their study, *LINE-1* methylation index was even higher in ADH than in normal breast tissue. The disagreement of their results from those of our study may be explained by not only different sample size, but also the different methods used in their study. In addition, DCIS is not homogeneous, but a heterogeneous group of diseases with diverse histologic features, molecular alterations and risks of progression to invasive cancer [31]–[33]. In our study, *LINE-1* methylation status was associated with ER and HER2 status and it was lowest in the HER2 enriched subtype in IBC. Although there were no differences in the *LINE-1* methylation levels with regard to ER and HER2 status in DCIS in this study (data not shown) probably due to the smaller sample size, the different proportion of ER-positive or HER2-positive DCIS in the samples may affect the results of *LINE-1* methylation levels. Further large-scale studies are needed to find if there is any difference in *LINE-1* methylation levels in regard to ER, HER2 and subtype status in DCIS. Moreover, it would be more reasonable to compare *Alu* and *LINE-1* methylation levels between DCIS and IBC according to the same grade or same ER or HER2 status, although we did not do so due to small sample size of DCIS.

*LINE-1* hypomethylation has been demonstrated as a poor prognostic maker in various human cancers such as colorectal [34], gastric [18], esophageal [35], and lung cancer [24], [36]. *Alu* hypomethylation has also been studied in human cancers, but its prognostic value is unclear in contrast to *LINE-1*. van Hoesel et al. reported that *LINE-1* hypomethylation was a negative prognostic factor for young breast cancer patients (≤55 years), and was associated with pT stage, lymph node metastasis, and higher age at diagnosis, but not with ER or HER2 status [23]. However, in this study, *LINE-1* hypomethylation was not associated with age at diagnosis, pT stage or lymph node metastasis. In contrast, it was associated with negative ER status, negative PR status, positive HER2 status, and p53 overexpression. Although *LINE-1* hypomethylation was associated with aggressive features of breast cancer such as negative ER status, positive HER2 status, and p53 overexpression, it was not associated with the disease outcome. These discrepancies may be caused by several factors, such as the use of different methods to measure methylation levels, racial difference, different follow-up periods, and different sample size. We investigated the methylation levels of *LINE-1* by using pyrosequencing, which is a validated method for quantifying methylation levels at individual CpG dinucleotides [18]. However, it cannot differentiate unmethylated CpG dinucleotides of partially methylated LINE-1s from unmethylated LINE-1s [30]. Thus, further genome-wide studies to measure the methylation levels of each LINE-1 locus are needed to verify the significance of *LINE-1* hypomethylation in breast cancer.

In this study, *Alu* and *LINE-1* methylation levels significantly differed among breast cancer subtypes, being lowest in HER2 enriched subtype. Isola et al. reported that *ERBB2 (HER2)* amplified breast cancers had a significantly higher number of chromosomal alterations, defined by comparative genomic hybridization, than HER2 non-amplified cancers [37]. Ellsworth et al. also showed that the frequency of allelic imbalance assessed by a panel of microsatellite markers representing 26 chromosomal regions was significantly higher in HER2 positive tumors than in HER2 negative tumors, suggesting global genomic instability in HER2 positive breast cancer [38]. Taken together, the prominent hypomethylation of *Alu* and *LINE-1* in the HER2 enriched subtype may be associated with chromosomal instability of this specific subtype. However, the difference in *Alu* and *LINE-1* methylation levels between luminal-HER2 and HER2 enriched subtypes cannot be explained by *ERBB2 (HER2*) amplification per se. The role of hormone receptor status in the *Alu* and *LINE-1* methylation status should be elucidated in further studies.

There seem to be genetic factors which affect *LINE-1* hypomethylation. Goel et al. reported that in Amsterdam criteria-fulfilled hereditary nonpolyposis colorectal cancer patients, tumors without mismatch repair deficiency are characterized by *LINE-1* hypomethylation [39]. Ogino et al. also showed that colorectal cancer family history is associated with a higher risk of *LINE-1* methylation-low colorectal cancer, suggesting unrecognized genetic predisposition to epigenetic alterations [40]. Recently, lower *LINE-1* methylation levels were demonstrated in white blood cell DNA of unaffected women with extensive breast cancer family history, indicating a role of epigenetic factors in familial clustering of breast cancer [41]. Thus, *LINE-1* methylation status may have clinical implication as a potential biomarker for cancer risk assessment in family members.

It was reported that CD44+ progenitor-like cells of normal mammary epithelium were globally hypomethylated compared to luminal epithelial (CD24+ and MUC1+) and myoepithelial (CD10+) cells and cell type-specific methylation patterns were conserved in breast cancer [42]. Thus, we expected that methylation levels of *Alu* and *LINE-1* may be different according to stem cell phenotypes. However, *Alu* and *LINE-1* methylation status did not show significant differences in relation to CD44+/CD24− phenotype, although they tended to be lower in tumors with CD44+/CD24− phenotype.

In summary, we have studied changes of *Alu* and *LINE-1* methylation levels during the multistep progression of breast cancer from normal to pre-invasive lesions and IBC, and the association of *Alu* and *LINE-1* methylation status with clinicopathologic features of IBC including breast cancer subtype, stem cell phenotype and disease outcome. *LINE-1* methylation was significantly decreased from normal to ADH/FEA, and *Alu* methylation tended to decrease during transition from DCIS to IBC. *Alu* and *LINE-1* methylation status was significantly different among breast cancer subtypes, being lowest in HER2 enriched subtype. *Alu* hypomethylation tended to be associated with poor disease-free survival of the patients and *LINE-1* methylation status was not associated with patients' survival. Our findings suggest that *LINE-1* hypomethylation is an early event and *Alu* hypomethylation is probably a late event during breast cancer progression, and that prominent hypomethylation of *Alu* and *LINE-1* in HER2 enriched subtype may be related to chromosomal instability of this specific subtype.

## Supporting Information

Figure S1 **Comparison of** ***LINE-1*** **methylation levels using paired samples of fresh frozen and formalin-fixed, paraffin embedded (FFPE) tissue.** A strong, positive linear correlation is observed between the two measures (Pearson correlation coefficient, 0.928; *P*<0.001).
(JPG)
