# Supplementary figures and images for: Alu and LINE-1 Hypomethylation Is Associated with HER2 Enriched Subtype of Breast Cancer
Source: PLoS One. 2014 Jun 27;9(6):e100429. doi: 10.1371/journal.pone.0100429 (PMC4074093; doi:10.1371/journal.pone.0100429)

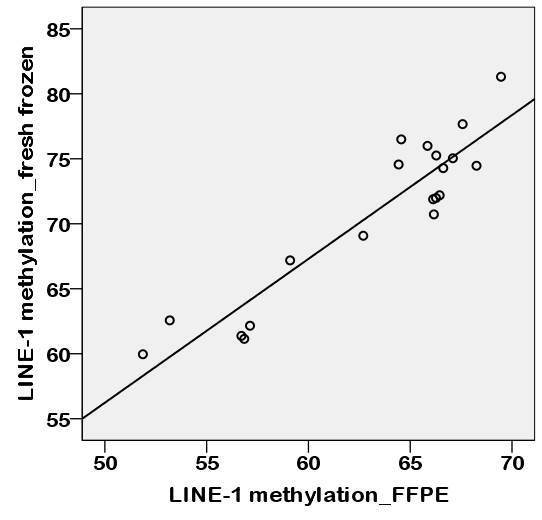

Supplement: Figure S1 — Comparison of LINE-1 methylation levels using paired samples of fresh frozen and formalin-fixed, paraffin embedded (FFPE) tissue. A strong, positive linear correlation is observed between the two measures (Pearson correlation coefficient, 0.928; P<0.001). (JPG) [file pone.0100429.s001.jpg]
